# The Last Days for Entering Insurance on Special Terms

**Published:** 1913-10-11

**Authors:** 


### NATIONAL INSURANCE ACT DEPARTMENT.

### LXX.—The Last Days for Entering Insurance on Special Terms.

The coming week is a very important one in connection with National Insurance, for on October 13 several of the chief alterations effected by the National Insurance Act, 1913, come into operation.

One of the concessions granted by the Act was the extension of time for entering insurance at full rates of benefit. This extended period expires on October 12, and as this is the last opportunity we would call the special attention of all whom it may concern to the advantages of entering insurance at once.

Two classes of persons are affected—namely, employed contributors and voluntary contributors. Most persons entitled to become employed contributors have already become insured; but there are even yet some few who should be paying contributions but are not doing so, and all such should take steps immediately to obtain the special advantages which are theirs only until October 12.

Every care was taken by the Insurance Commissioners and by the approved societies when the Act of 1911 came into operation in July 1912 to bring to the notice of employers and workers alike the requirements of the Act. These efforts, with the peaceful persuasion of inspectors and very rare prosecutions for non-compliance, resulted in the vast majority of eligible employed persons securing for themselves the advantages conferred by the Act. In spite of all this propaganda, it was realised when the Amendment Bill was under consideration that there were a comparatively small number of persons, engaged for the most part in domestic employments on a small scale, who had not complied with the requirement to become insured, and had thereby lost the special privileges attaching to entry within the first year of the operation of the Act. For this reason, among others, the period was extended by three months in order to provide a further opportunity of obtaining the full benefits of the Act for those who, by inadvertence or other cause, had not become insured.

### The Advantage of Immediate Entry.

The concession is in many cases a very valuable one, for it means that everyone entering insurance for the first time is entitled, no matter what his age may be, to the benefits applicable to a person of the same sex aged sixteen years at entry, while the contribution is the same.

It is not commonly known how great an advantage this is. Its actual value will, of course, differ with every individual. There may be some, in fact, who will not benefit by it at all—those, for instance, who are so fortunate as never to be incapacitated from work by illness until they pass out of insurance altogether. On the other hand, those who by prolonged illness receive much more than the average amount of sick pay will benefit very largely. Taking one with another, on the insurance system the value varies according to the age at entry. At some ages it is actually worth more than a cash present of £10 on the basis of the actuarial estimates, and for all ages above thirty-two in the case of men it is worth more than £5. Similar values apply in the case of women. Few indeed of those who are entitled to become employed contributors can be in a position to ignore such advantages, and if they are to be obtained it must be at once.

### How to Become Insured.

Fortunately, it is not difficult to secure entry into insurance. All that has to be done is to obtain an insurance card, either from a post office or from an approved society, and affix a special Insurance stamp, which should immediately be cancelled, with the date of cancellation clearly marked. Provided this is done on or before October 12, entry has been made in time, though it will, of course, be necessary to proceed to gain admittance as a member of an approved society if the full benefits of insurance are to be received.

It should not be thought by anyone that by deferring entry he will be relieved of the obligation of insurance imposed by the Act. The resister, if any such remain, will undoubtedly be discovered in course of time, and will then find that, instead of being entitled to sickness benefit at the rate of 10s. a week in the case of men and 7s. 6d. in the case of women, the benefit will be at the reduced rate applicable to the age at entry, and may be less than half the sums just mentioned.

To all employed persons above sixteen years of age who are not receiving remuneration at a higher rate than £160 a year and are not otherwise excepted from the Act, the writer advises immediate action to obtain the advantages conferred on those who enter within the first sixty-five weeks after the Act came into operation. This advice extends to those employed contributors who have obtained exemption certificates. Although, under the provisions of the Amendment Act, exempted persons are entitled to receive medical and sanatorium benefits in consideration of the contributions paid by their employers, there appear to be few cases where the exempted person would not be well advised to pay his own contribution and thereby secure his right to the sickness, disablement, and maternity benefits.

### The Concession to Voluntary Contributors.

The advantages to voluntary contributors are much are same as those above described for employed persons. In the case of voluntary contributors the Act of 1911 allowed six months only as the period during which entry could be made into insurance at special rates. That period expired on January 15 last. The extension of the time to October 12 by the Amendment Act was therefore an even greater concession than that given to employed persons.

The voluntary contributor class is composed of those who, while not employed within the meaning of the Act, are engaged in an occupation on their own account, and whose total income from all sources is not more than £160 per annum. It was thought that many such persons would be glad to avail themselves of the advantages of insurance, but in this respect the estimates have not been fulfilled. This may have been due in some measure to the political agitation against the Act, but in any case the fact remains. The extra time was undoubtedly given to enable those to take advantage of the Act who in its early stages and before the benefits became payable had had little opportunity of gauging the value that insurance against sickness would be to them. The principal difference between the classes of employed and voluntary contributors in regard to deferred entry into insurance is that, while in the former case the contribution is maintained at 7d. a week for men and 6d. a week for women, and the benefits are made to correspond thereto, in the latter class the benefits will remain the same, but a higher weekly contribution will be required. Thus, for instance, a male voluntary contributor under forty-five years of age at entry into insurance not later than October 12 will pay 7d. a week, but if entry be deferred until after that date and his age is forty-four years the weekly contribution will be 10½d. This is an increase of 50 per cent. in the weekly contribution, and it is clear that the advantage of the lower rate is well worth having. The principle involved is exactly the same in the case of the voluntary contributor as of the employed contributor, but the difference in its application enables another view to be taken of the matter, and together overwhelming reasons are shown for taking advantage of the opportunity that only extends until October 12.

